# PAFR activation of NF-κB p65 or p105 precursor dictates pro- and anti-inflammatory responses during TLR activation in murine macrophages

**DOI:** 10.1038/srep32092

**Published:** 2016-08-24

**Authors:** Edson K. Ishizuka, Luciano Ribeiro Filgueiras, Francisco J. Rios, Carlos H. Serezani, Sonia Jancar

**Affiliations:** 1 Department of Immunology, Institute of Biomedical Sciences, University of São Paulo, São Paulo, Brazil; 2 Institute of Cardiovascular and Medical Sciences, BHF Glasgow Cardiovascular Research Centre, University of Glasgow, Glasgow, United Kingdom; 3 Department of Microbiology and Immunology, Indiana University School of Medicine, Indianapolis, Indiana, (46202) United States of America

## Abstract

Platelet-activating factor receptor (PAFR) is a G protein-coupled receptor (GPCR) implicated in many diseases. Toll-like receptors (TLRs) play a critical role in shaping innate and adaptive immune responses. In this study, we investigated whether PAFR signaling changes the macrophages responsiveness to agonists of TLR2 (Pam3Cys), TLR4 (LPS), and TLR3 agonist Poly(I:C). Exogenous PAF inhibited the production of pro-inflammatory cytokines (IL-12p40, IL-6, and TNF-α) and increased anti-inflammatory IL-10 in macrophages challenged with Pam3Cys and LPS, but not with Poly (I:C). PAF did not affect mRNA expression of MyD88, suggesting that PAF acts downstream the adaptor. PAF inhibited LPS-induced phosphorylation of NF-κB p65 and increased NF-κB p105 phosphorylation, which is processed in the proteasome to generate p50 subunit. The PAF potentiation of IL-10 production was dependent on proteasome processing but independent of NF-κB transactivation domain. Inhibition of p50 abolished the PAF-induced IL-10 production. These findings indicate that the impaired transcriptional activity of the p65 subunit and the enhanced p105 phosphorylation induced by PAF are responsible for down regulation of pro-inflammatory cytokines and up regulation of IL-10, respectively, in LPS-challenged macrophages. Together, our data unveil a heretofore unrecognized role for PAFR in modulating activation of NF-κB in macrophages.

Macrophages are fundamental to almost all aspects of organismal biology, ranging from development to homeostasis, tissue repair, clearance of dying cells, and resistance to infections^1^. These functions of macrophages depend on their capacity to recognize molecular patterns that are conserved in microbial species, known as pathogen-associated molecular patterns (PAMPs), as well as moieties present in altered *self,* including oxidized lipids in dying cells or molecules released during trauma and cell stress^2^.

Recognition depends on a wide range of receptors, which include the Toll-like and “scavenger” receptor families^3^,^4^. Stimulation of these receptors promotes activation of phospholipase A2, which cleaves polyunsaturated fatty acids from membrane phospholipids to generate eicosanoids and PAF (1-*O*-alkyl-2-acetyl-*sn*-glycero-3-phosphocoline)^5^. PAF acts on its cognate receptor (PAFR), a G protein-coupled receptor (GPCR) cloned in 1991 by Honda *et al*., that couples to the subunits Gα_q_ and Gα_I,_ and promotes cell activation with calcium, cAMP, IP_3_, and diacylglycerol being second messengers^6^.

There is evidence of a PAFR association with the recognition receptor CD36. Work from our laboratory has shown that concomitant activation of CD36 and PAFR is required for optimal oxidized LDL uptake or phagocytosis of apoptotic cells by murine and human macrophages. These receptors co-immunoprecipitate with markers for *lipid rafts*, flotilin-1 and GM1, suggesting that receptor association occurs in these membrane microdomains. It was also shown that both receptors are required for downstream activation, gene transcription, and cytokine production. Moreover, the presence of PAFR is associated with an anti-inflammatory profile characterized by high production of interleukin-10 (IL-10) and low levels of IL-12 by macrophages^7^,^8^. These macrophages are identified as regulatory macrophages, as proposed by Fleming & Mosser^9^ and have an important function in the clearance of apoptotic cells and a “damping” effect on the inflammatory process^8^.

Toll-like receptors (TLRs) play a critical role in host defense against infectious agents. Approximately 13 mammalian TLRs have been identified in murine cells and 10 in humans^10^. Although TLRs share many proteins for cell signaling activation, each one exerts some particular effects, which are attributed to different adaptors, co-receptors, and types of receptor dimers. MyD88 (myeloid differentiation factor 88) and TRIF (TIR-domain-containing adaptor protein inducing IFNβ) are different Toll/interleukin-1 receptor (TIR) adaptor proteins. While MyD88 is utilized by most TLRs except TLR3, TRIF is used by both TLR3 and TLR4. Both adaptors induce molecular pathways that culminate in the activation of transcription factors, MyD88 being more efficient and faster than TRIF in activating NF-κB (nuclear factor kappa B)^11^,^12^.

NF-κB plays critical roles in inflammation, survival, stress response, and the cell cycle^13^,^14^,^15^,^16^. There are five NF-κB family members in mammals: RelA/p65, RelB, c-Rel, p50 (NF-κB1), and p52 (NF-κB2). Different dimer combinations of these subunits can be formed and the heterodimer p50/p65 is classically known to induce pro-inflammatory gene expression^17^. In non stimulated cells, p50/p65 NF-κB is sequestered in the cytoplasm by the inhibitor protein IκBα and upon stimulation, it undergoes phosphorylation and degradation in the proteasome, leading to the release of the p50/p65 heterodimer, nuclear translocation, and DNA binding, leading to gene transcription^18^,^19^.

Given the fact that PAFR drives anti-inflammatory programs in macrophages when associated with the scavenger receptor CD36^7^, we asked what effect PAFR would have on the pro-inflammatory program elicited by TLRs. We speculated that PAFR affect different subunits of NF-κB to induce pro- and anti-inflammatory cytokines. Here, we examined the effect of PAF on the inflammatory program elicited by agonists of TLRs, MyD88-dependent Pam3Cys (TLR2), LPS (TLR4), and the MyD88-independent Poly (I:C) (TLR3) in murine macrophages. We found that PAF inhibits production of pro-inflammatory mediators induced by TLR2 and TLR4 and enhances IL-10 levels, promoting an anti-inflammatory phenotype. We also identified that PAF acts on the NF-κB p65 subunit to decrease pro-inflammatory cytokines and on p105/50 to induce the anti-inflammatory IL-10. Overall, we show that PAF interferes with the NF-κB pathway induced by TLRs in macrophages, driving them towards a regulatory phenotype.

## Materials and Methods

### Animals

Male C57BL/6 mice at 6 to 8 weeks of age from the Biomedical Sciences Institute at the University of São Paulo were used. All experiments were carried out according to the guidelines provided by the Brazilian College of Animal Experimentation and approved by the Animal Ethics Committee of the Biomedical Sciences Institute, University of São Paulo (129/67-12).

### Reagents and antibodies

TLR agonists LPS from *Escherichia coli* serotype 026:B6 (TLR4), Pam3Cys (TLR2) and Poly(I:C) (TLR3) were purchased from Sigma (St. Louis, MO), Calbiochem (San Diego, CA), and Invivogen (San Diego, CA), respectively. Methylcarbamyl PAF C-16 (cPAF, a more metabolically stable analog) was purchased from Cayman Chemical (Ann Arbor, MI). NF-κB inhibitors acetyl-L-leucyl-L-leucyl-L-norleucinal (ALLN) and pyrrolidinedithiocarbamate (PDTC) were obtained from Tocris (Minneapolis, MN). CREB inhibitor KG-501 (2-naphthol-AS-E-phosphate) and PPARγ antagonist GW9662 were obtained from Sigma (St. Louis, MO). NF-κB p105/p50 inhibitor peptide was obtained from Novus Biologicals (Littleton, CO). Anti-COX2 Ab was supplied by Cayman (Ann Arbor, MI). Anti-iNOS, anti-phospho-IκBα (Ser32), anti-phospho-NF-κB p65 (Ser536), anti-phospho STAT3 (Tyr705), anti-β-actin, and secondary Abs goat anti-rabbit IgG and goat anti-mouse IgG conjugated to HRP were purchased from Cell Signaling Technology (Danvers, MA). Anti phospho-NF-κB p105/p50 (Ser337) was from SAB Signaling (College Park, MD).

### Macrophage isolation and stimulation

Elicited macrophages were harvested from the peritoneal cavity of mice by lavage with cold PBS four days after i.p. injection of 4% Brewer thioglycolate medium (Difco, Surrey, UK). After isolation, the macrophages were resuspended in RPMI 1640 (Life Technologies, Carlsbad, CA) and allowed to adhere to tissue culture-treated plates for 1 h at 37 °C and 5% CO_2_. Cells were then washed twice with pre warmed PBS and cultured overnight in RPMI containing 10% fetal bovine serum (Gibco, Grand Island, NY). On the next day, macrophages were stimulated concomitantly with cPAF (100 nM)^7^ and LPS (100 ng/mL), Pam3Cys (100 ng/mL) or Poly(I:C) (50 μg/mL) for different time periods (4, 8, and 24 h). In some experiments, macrophages were pretreated with ALLN (1, 10 and 30 μM), PDTC (25, 50 and 100 μM), KG-501 (3, 10, and 100 μM) or GW9662 (10 μM) for 30 min, or NF-κB p105/p50 inhibitor peptide (25 μM) for 60 min prior to cPAF and TLR agonists stimulation for 8 h.

### MTT assay

A total of 2 × 10^6^ macrophages were plated in 12-well flat bottom plates and then stimulated with cPAF and LPS. After 8 h, the supernatants were removed and 500 μL of 5 mg/mL MTT solution in RPMI were added to each well for 4 h. After removal of the medium, 200 μL of DMSO were added to each well to dissolve the formazan crystals. The absorbance at 540 nm was determined using a spectrophotometer.

### Measurement of cytokines

Production of IL-12p40, IL-6, TNF-α, and IL-10 in the supernatant of the macrophage culture was measured using OptEIA^TM^ Mouse Set ELISA kits (BD Pharmingen, San Diego, CA) according to the manufacturer’s instructions.

### PGE_2_ quantification

PGE_2_ production was measured in the supernatants of macrophage cultures by competitive immunoassay using a PGE_2_ EIA kit (Cayman Chemical, Ann Arbor, MI) according to the manufacturer’s instructions.

### RNA isolation and quantitative PCR (qPCR)

Total RNA was extracted from cultured cells using TRIzol reagent (Ambion Life Technologies, Carlsbad, CA) and the concentration and purity of the samples were determined by spectrophotometer readings at 260 nm and 280 nm. Single-stranded cDNA was synthesized using the RevertAid First Strand cDNA Synthesis kit (Thermo Scientific, Rockford, IL). The following PCR primers were used: *Myd88* (**F**: 5′-TGA TGA CCC CCT AGG ACA AA-3′; **R**: 5′-TCA TCT CCT GCA CAA ACT CG-3′) and *GAPDH* (**F**: 5′-AGG TCG GTG TGA ACG GAT TTG-3′; **R**: 5′-TGT AGA CCA TGT AGT TGA GGT CA-3′). Real-time qPCR was performed in a SYBR Green PCR Master Mix (Applied Biosystems, Life Technologies, Warrington, UK) using the Stratagene Mx3005P qPCR System with the following cycling conditions: initial denaturation and enzyme activation for 10 min at 95 °C, followed by 40 cycles of 95 °C for 15 s and 60 °C for 1 min. Data were normalized to *GAPDH* expression and the relative abundance of transcripts was calculated by the comparative 2^−ΔΔC^_T_ method as described previously^20^.

### Immunoblotting

Whole macrophage lysates containing 30 μg of protein were separated by 10% SDS-PAGE and transferred to polyvinylidene difluoride (PVDF) membranes (GE Healthcare, Little Chalfont, Buckinghamshire, UK). Next, non-specific binding sites were blocked with 5% non-fat dry milk in TBS-T (150 mM NaCl, 20 mM Tris, 0.5% Tween 20, pH 7.4) for 1 h. After washing with TBS-T, the blots were incubated with primary antibodies against COX2 (1:400), phospho-IκBα Ser32 (1:1000), phospho-NF-κB p65 Ser536 (1:1000), phospho-STAT3 Tyr705 (1:000), phospho-NF-κB p105/p50 Ser337 (1:500) or β-actin (1:1000) at 4 °C overnight. After that, the blots were washed and incubated with secondary antibodies goat anti-rabbit IgG or goat anti-mouse IgG conjugated to HRP (1:3000) for 2 h. Finally, the membranes were developed using an enhanced chemiluminescent substrate (Thermo Scientific, Rockford, IL). Densitometric analysis were performed using Alpha Digidoc RT2 imaging software (Alpha Innotec, San Leandro, CA) and represented as arbitrary unit (A.U.) relative to control group.

### Statistical analysis

Data are represented as mean ± SD and were analyzed using the Prism 5.0 statistical program (GraphPad Software, San Diego, CA). Comparisons among groups were performed by ANOVA followed by the Bonferroni multiple comparison test. A *P* value ≤ 0.05 was considered to indicate a statistically significant result.

## Results

### PAF modulates the production of inflammatory cytokines induced by TLR2 and TLR4 in macrophages

**Figure 1:**
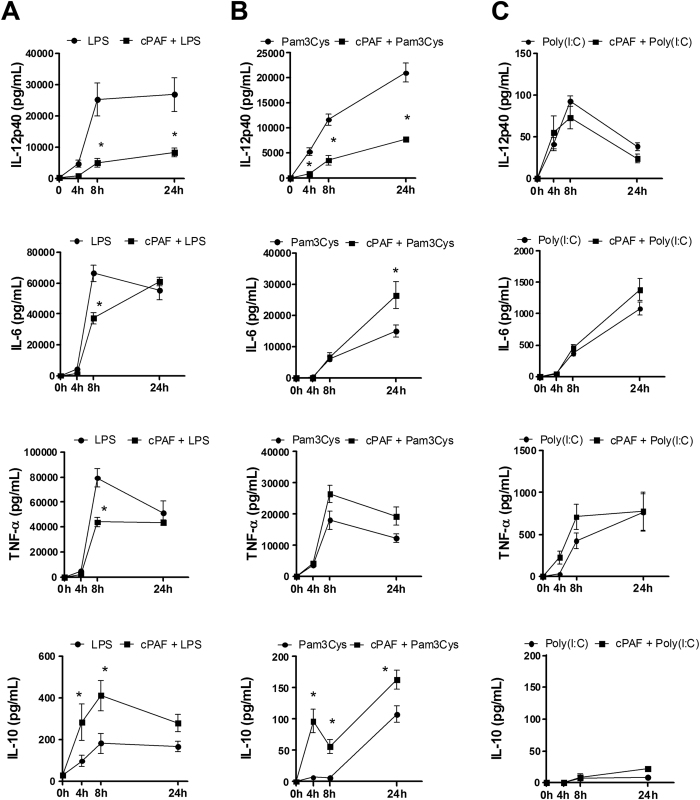
PAF modulates inflammatory cytokine production induced by TLR2 and TLR4. Peritoneal macrophages isolated from C57BL/6 mice were stimulated with (**A**) LPS 100 ng/mL, (**B**) Pam3Cys 100 ng/mL or (**C**) Poly(I:C) 50 μg/mL agonists alone or in combination with cPAF 100 nM. After 4, 8, and 24 h, the supernatants were collected and IL-12p40, IL-6, TNF-α, and IL-10 concentrations were measured by ELISA. The data are representative of three independent experiments and presented as mean ± SD of n = 5 animals per group. *p < 0.05 compared with LPS or Pam3Cys.

Initially, to determine the role of PAF in TLR-induced macrophages response, we challenged macrophages with PAF plus the TLR agonists LPS (TLR4), Pam3Cys (TLR2) and Poly(I:C) (TLR3). Our results show that PAF signaling inhibited both LPS and Pam3Cys-induced production of IL-12p40 after both 8 and 24 h of exposure to stimuli, whereas IL-6 and TNF-α levels were decreased after 8 h of stimulation by LPS. However, PAF potentiated the production of IL-10 induced by both agonists at both time points tested (Fig. 1A,B). However, PAF did not affect Poly(I:C) -induced cytokine production (Fig. 1C), indicating that PAF actions on TLR activation are restricted to TLR2 and TLR4.

**Figure 2:**
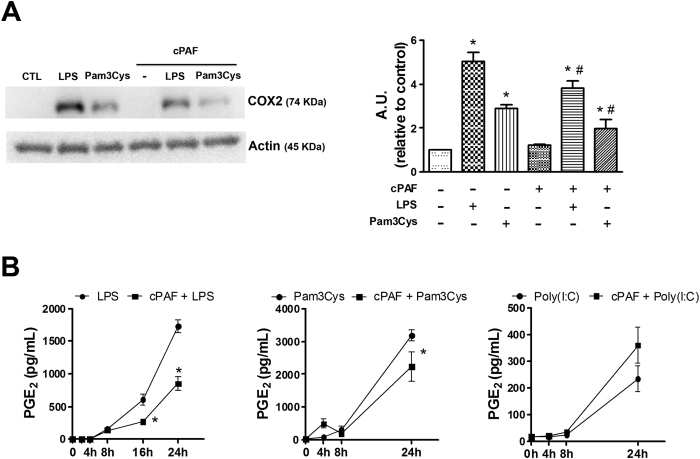
Effect of PAF on NF-κB-dependent COX2 expression induced by TLR agonists. Peritoneal macrophages isolated from C57BL/6 mice were stimulated with LPS 100 ng/mL or Pam3Cys 100 ng/mL alone or in combination with cPAF 100 nM. (**A**) Lysates obtained after 24 h of stimulation were evaluated for COX2 by immunoblotting. Cropped blot is shown from one representative experiment. Full-length gels are included in the Supplementary information file. Densitometric analysis of COX2 protein levels is represented as mean ± SD from 5 independent experiments. (**B**) Supernatants from macrophages cultured for the indicated times were subjected to PGE_2_ quantification. For PGE_2_ quantification, the data represent three independent experiments and are presented as mean ± SD of n = 5 animals per group. *p < 0.05 compared with control, ^#^*p* < 0.05 compared with LPS group. A.U., arbitrary units.

In addition to changes in gene transcription, PAF could influence the activity of enzymes involved in macrophage activation, such as cyclooxygenase 2 (COX2), which generates different prostanoids, including prostaglandin E_2_ (PGE_2_). As demonstrated in Fig. 2A, concomitant treatment of macrophages with PAF plus LPS or Pam3Cys inhibited COX2 expression after 24 h of stimulation as compared with either agonist alone. Furthermore, these findings correlated with decreased PGE_2_ accumulation in macrophage cultures stimulated as above (Fig. 2B).

### PAF does not influence *Myd88* expression in TLR-stimulated macrophages

**Figure 3:**
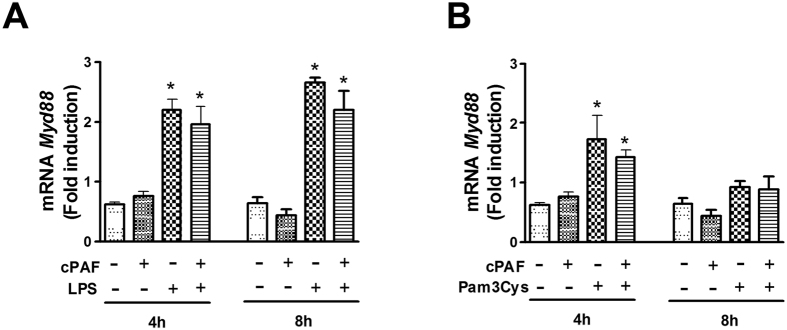
PAF does not affect TLR-induced *Myd88* gene expression. Total RNA samples isolated from macrophages stimulated with LPS 100 ng/mL, Pam3Cys 100 ng/mL, or Poly(I:C) 50 μg/mL alone or combined with cPAF 100 nM for the indicated times were subjected to qPCR and the *Myd88* mRNA expression induced by (**A**) LPS and (**B**) Pam3Cys was evaluated. The results are representative of one independent experiment. Data are mean ± SD of n = 4 animals per group. *p < 0.05 compared with control.

To further investigate the downstream effects of PAF on TLR activation, we studied whether PAF inhibits the expression of *Myd88* mRNA, which could consequently affect cytokine production induced by LPS and Pam3Cys. Our results show that while both LPS and Pam3Cys enhance *Myd88* expression, PAF did not influence TLR-enhanced Myd88 expression (Fig. 3A,B), suggesting that PAF influences signaling effectors downstream of MyD88 actions during TLR activation.

### PAFR differentially regulates NF-κB subunits to modulate TLR-induced cytokine generation

**Figure 4:**
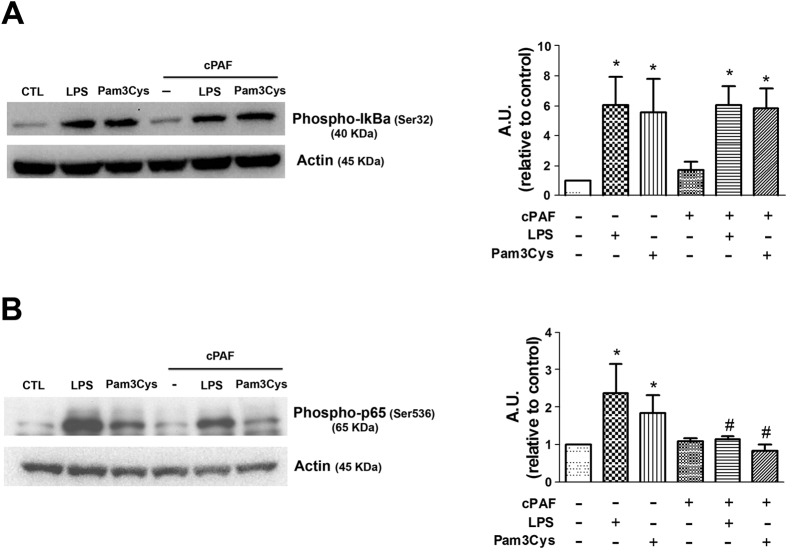
Effect of PAF on TLR2/4–mediated IκBα and p65 phosphorylation. (**A**) Peritoneal macrophages isolated from C57BL/6 mice were stimulated with LPS 100 ng/mL or Pam3Cys 100 ng/mL alone or in combination with cPAF 100 nM. Lysates obtained after 60 and 15 min of stimulation were probed with antibodies against (**A**) phospho-IκBα and (**B**) phospho-NF-κB p65, respectively. Cropped blots are shown from one representative experiment. Full-length gels are included in the Supplementary information file. Densitometric analysis of phospho-IκBα and phospho-p65 protein levels is represented as mean ± SD from 5 independent experiments. *compared with control; ^#^*p* < 0.05 compared with LPS group. A.U., arbitrary units.

Next, we sought to study the signaling programs downstream of MyD88 that are involved in PAF effects on TLR activation. Since NF-κB activation drives both pro- and anti-inflammatory networks, we speculated that PAF could differentially influence NF-κB dimer formation. Initially we investigated the effect of PAF on inhibitory protein IκBα expression and did not observe any effect of PAF, neither in LPS nor in Pam3Cys-mediated IκBα activation (Fig. 4A), indicating that the effects of PAF could be downstream of changes in IκBα degradation. Another regulatory level of NF-κB activation is p65 phosphorylation, an event required for optimal NF-κB transcriptional activity^21^. As seen in Fig. 4B, PAF decreased the phosphorylation of p65 induced by LPS in 15 min, indicating that PAF inhibits the production of pro-inflammatory cytokines by affecting p65 transcriptional activity.

**Figure 5:**
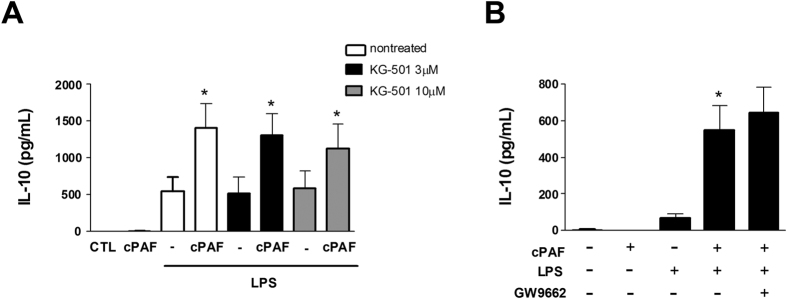
PAF-induced IL-10 increase is not dependent on CREB or PPAR-γ. Peritoneal macrophages were pretreated with (**A**) CREB inhibitor KG-501 for 1 h or (**B**) PPAR-γ antagonist GW9662 for 30 min. Cells were then stimulated with LPS 100 ng/mL and cPAF 100 nM at the same time. After 8 h, culture supernatants were collected and IL-10 levels determined by ELISA. The data are representative of one independent experiment and presented as mean ± SD of n = 5 animals per group. *p < 0.05 compared with control.

In order to identify the molecular pathways involved in the PAF-mediated effects, we enquired whether other transcription factors and receptors involved in IL-10 production, such as CREB (cAMP response element binding protein) and PPAR-γ (peroxisome proliferator-activated receptor-γ)^22^,^23^, could be involved in PAF-induced anti-inflammatory programs during TLR activation. Neither CREB (KG-501) nor PPAR-γ (GW9662) inhibitors prevented PAF-enhanced IL-10 production (Fig. 5A,B).

**Figure 6:**
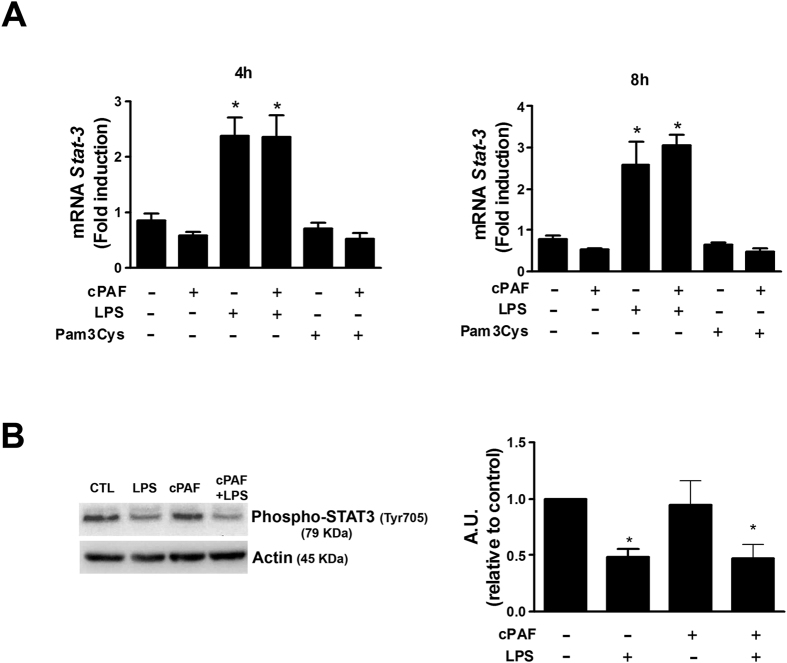
Expression of STAT3 in macrophages stimulated with PAF and TLR2 and TLR3 agonists. (**A**) Peritoneal macrophages were exposed to LPS 100 ng/mL or Pam3Cys 100 ng/mL alone or combined with cPAF 100 nM. At the indicated times, total RNA was isolated and samples were probed for *Stat3* mRNA levels by qPCR. (B) Protein samples were incubated with Ab to phosphorylated STAT3. Cropped blot is shown from one representative experiment. Full-length gels are included in the Supplementary information file. Densitometric analysis of STAT3 protein levels is represented as mean ± SD from 5 independent experiments. For qPCR, the data are representative of one independent experiment and presented as mean ± SD of n = 5 animals per group. *p < 0.05 compared with control. A. U., arbitrary units.

Furthermore, when we investigated whether PAF could change the phosphorylation of STAT3 (which drives IL-10 expression and action) we did not observe changes in STAT3 expression and activation during PAF + LPS co-incubation (Fig. 6A,B).

We next investigated whether NF-κB activation was involved in PAF-induced IL-10 potentiation. We first tested the proteasome inhibitor Calpain inhibitor acetyl-L-leucyl-L-leucyl-L-norleucinal (ALLN), that prevents the proteolysis of IκBα and IκBβ by the ubiquitin-proteasome complex^24^.

**Figure 7:**
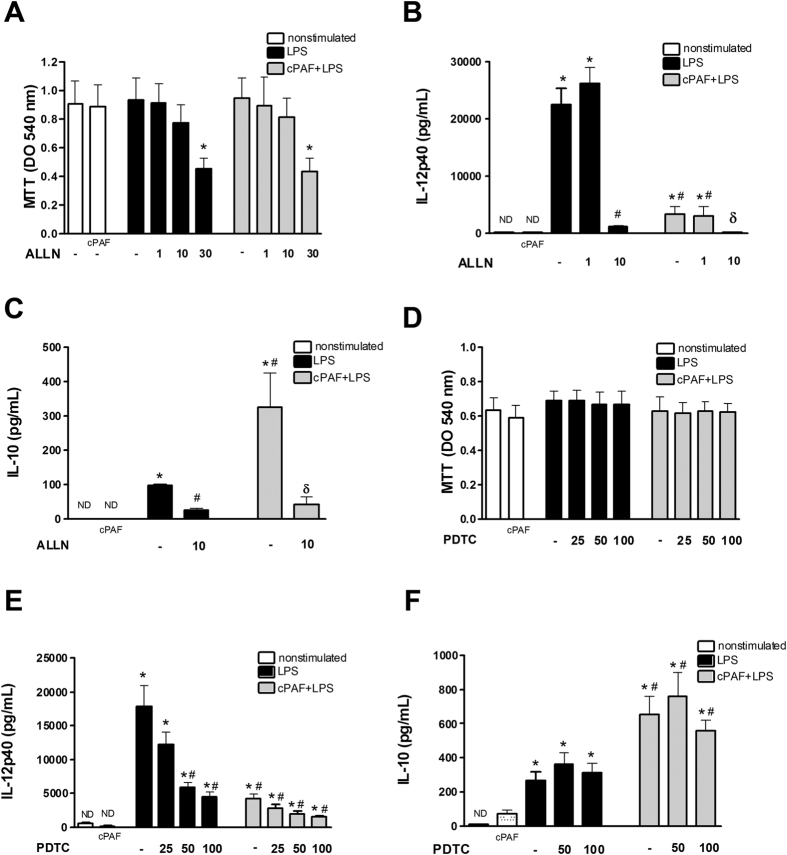
Effect of NF-κB inhibitors on PAF-mediated IL-12p40 and IL-10 modulation. Peritoneal macrophages were pretreated with NF-κB inhibitors ALLN (1, 10 and 30 μM) or PDTC (25, 50 and 100 μM) for 30 min and then exposed to LPS (100 ng/mL) and cPAF (100 nM). After 8 h, the cells were submitted to MTT assay (**A,D**) and the levels of IL-12p40 and IL-10 in the macrophage cultures supernatants pretreated with ALLN (**B,C**) and PDTC (**E,F**) were measured by ELISA. The data are representative of one independent experiment and presented as mean ± SD of n = 5 animals per group. *p < 0.05 compared with control. ^#^*p* < 0.05 compared with LPS. ^δ^p < 0.05 compared with cPAF + LPS.

The ALLN treatment did not affect macrophages viability at 1 and 10 μM, measured by MTT assay (Fig. 7A). When proteasome activity was abrogated, indicated by IL-12 inhibition at 10 μM (Fig. 7B), the IL-10 production was also inhibited (Fig. 7C), suggesting that IL-10 production is dependent on proteasome processing.

We next tested the pyrrolidinedithiocarbamate (PDTC) treatment, that inhibits the transactivation domain of NF-κB subunits^25^. The PDTC treatment did not affect cell viability at all tested doses (Fig. 7D). At 50 and 100 μM, PDTC inhibited IL-12 production (Fig. 7E) indicating that it effectively inhibited the transactivation domain, but did not affect PAF-induced IL-10 production (Fig. 7F), suggesting that the transactivation domain is not involved in PAF-induced IL-10 potentiation triggered by LPS.

**Figure 8:**
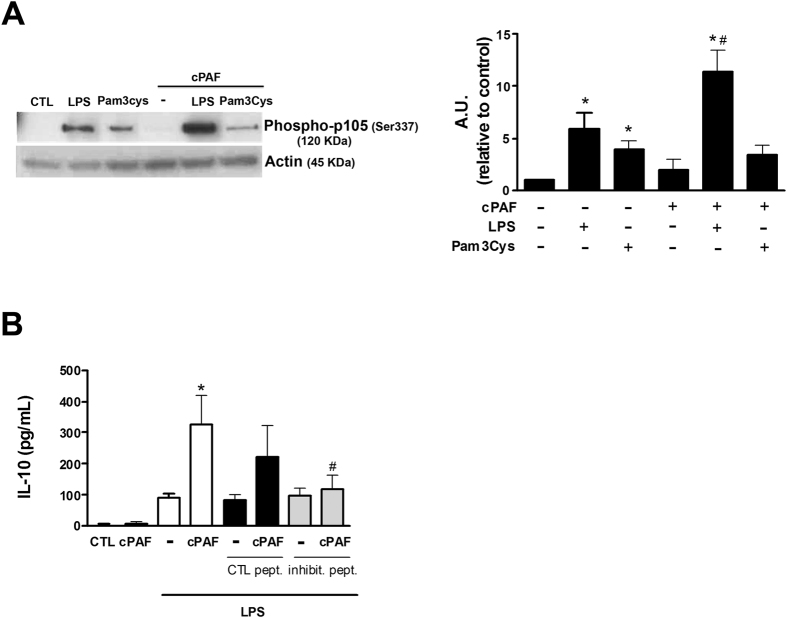
PAF potentiates LPS-induced IL-10 by affecting NF-κB p105 precursor. Peritoneal macrophages were simultaneously exposed to LPS (100 ng/mL) and cPAF (100 nM). (**A**) After 15 min of stimulation, cell lysates were subjected to immunoblotting using specific Ab against phosphorylated NF-κB p105/p50. Cropped blot is shown from one representative experiment. Full-length gels are included in the Supplementary information file. Densitometric analysis of p105 protein levels is represented as mean ± SD from 5 independent experiments. (**B**) Macrophages pretreated for 30 min with NF-κB p105/p50 inhibitor or peptide control were stimulated with LPS 100 ng/mL and cPAF 100 nM. After 8 h, the levels of IL-10 in the culture supernatants were measured by ELISA. The data are representative of one independent experiment and presented as mean ± SD of n = 5 animals per group. *p < 0.05 compared with control. ^#^*p* < 0.05 compared with cPAF + LPS. A.U., arbitrary units.

The transactivation domain is present in the p65, c-Rel and Rel B subunits of NF-κB, but not in p100/52 and p105/p50^17^. NF-κB p105/p50 is known to enhance the IL-10 transcriptional machinery^26^. Therefore, we speculated that PAF acts on NF-κB p105/p50, increasing TLR-induced IL-10. Our data show that PAF enhances LPS phosphorylation of the p105 subunit compared with LPS alone (Fig. 8A), but the same was not observed with Pam3Cys. To confirm that p105 is implicated in the IL-10 increase induced by PAF + LPS treatment, macrophages were pretreated for 1 h with a p105/p50 inhibitory peptide followed by LPS stimulation for 8 h. Our data show that p105/p50 inhibition, but not the peptide control, reduced PAF-mediated IL-10 increase (Fig. 8B).

Taken together, our results imply that PAF drives pro- and anti-inflammatory responses by controlling p65 and p105 NF-κB subunits phosphorylation during TLR activation in macrophages.

## Discussion

Here we investigated the effect of PAFR activation on macrophage responsiveness to TLR agonists. In summary, our results show that PAF reduces the generation of pro-inflammatory cytokines and PGE_2_ and increases the generation of anti-inflammatory IL-10 in macrophages stimulated with LPS or Pam3cys. Similar results were described by Jeong *et al*.^27^ in LPS-stimulated murine peritoneal macrophages where PAF was shown to potentiate IL-10 production and inhibit IL-12, IL-6, and TNF-α. IL-10 is the prototypic anti-inflammatory cytokine that acts by binding to the IL-10 receptor present on various cell types^28^. Mice genetically deficient in the IL-10 receptor develop an exacerbated inflammatory response and autoimmune diseases. Impaired IL-10 responses have been linked to human diseases, including inflammatory bowel diseases, arthritis, asthma, and psoriasis^29^,^30^,^31^. PAFR KO mice also exhibit sterile inflammation and insulin resistance by increasing pro-inflammatory macrophages in the adipose tissue^32^. Thus, the PAF/IL-10 axis might have an important role in preserving homeostasis by down regulating inflammation.

In our study, PAF induced a regulatory phenotype (IL-10^high^/IL-12^low^) in both LPS and Pam3Cys-stimulated macrophages. Regulatory macrophages are known to modulate inflammatory responses. Ziegler *et al*.^33^ demonstrated that adoptive transfer of regulatory macrophages increases local and systemic IL-10 production while it attenuates allergic airway inflammation, decreasing allergen-specific IgE levels, eosinophil influx to the airways, Th2 cytokine production, and the production of mucus in the lungs. A regulatory phenotype was also observed in FcγR-activated macrophages that were able to reduce lethal endotoxemia by preventing pro-inflammatory cytokine responses^34^.

The PAF effects on the regulatory phenotype are not limited to macrophages, as similar effects were observed in LPS-stimulated murine dendritic cells, potentiating IL-10 and reducing pro-inflammatory cytokines and, in addition, downregulating antigen-presenting function^35^. We also recently found that transfer of dendritic cells that were treated with a PAFR antagonist increased antigen-specific lymphocyte proliferation^35^. Based on these and other line of evidence, we propose that activation of PAFR by endogenous ligands might be important to the “fine-tune” regulation of inflammatory and immune responses. As endogenous ligands, several oxidized phospholipids, including those expressed in apoptotic cell plasma membranes, bind to PAFR^36^. Also, UV radiation and several environmental hazards were shown to induce the production of molecules that bind to PAFR^37^. Chemo- and radiotherapy also induce PAFR ligands and this was shown to promote tumor growth, in part because they induce regulatory macrophages^38^.

The dual effect of PAF, inhibition of pro-inflammatory cytokines and potentiation of the anti-inflammatory IL10, was dependent on the inhibition of NF-κB p65 phosphorylation and enhancement of NFκB p105/50 phosphorylation, respectively. We have previously shown that the LTB_4_ receptor BLT1, a receptor that is coupled to Gα_i_ protein, potentiates MyD88-dependent TLR activation, but not TRIF-dependent activation^39^,^40^. In the present study, PAF did not affect MyD88 expression, indicating that the PAF effects on pro-inflammatory cytokine production are downstream of adaptors.

We found that PAF does not affect TLR-induced IκBα phosphorylation, but inhibits NF-κB p65 phosphorylation at serine 536. Different GPCR subunits are known to activate NF-κB. Plasma membrane-associated PAFR is coupled to Gα_q_ and activates NF-κB. Bradykinin receptor 2 utilizes a signaling pathway that involves Gα_q_ for NF-κB activation by enhancing IκBα phosphorylation^41^. The receptor for FMLP (N-formyl-Met-Leu-Phe), coupled to Gα_I_ also activates NF-κB in neutrophils^42^. Interestingly, PGE_2_ also activates NF-κB via the Gα_s_-coupled receptors, EP2 and EP4, in a PKA-dependent manner^43^. However, whether different G proteins coupled to PAF receptors influence different pathways that culminate in pro- or anti-inflammatory events remain to be investigated.

It is well documented that the IKK complex, which triggers IκBα degradation, is involved in p65 phosphorylation^44^. Yang *et al*.^45^ described an essential role of IKKβ in LPS-induced S536 p65 phosphorylation and, according to our results, this phosphorylation is reduced by PAF treatment. Thus, PAF could influence the actions of phosphatases involved in p65 activation in a similar manner as described for WIP-1, a p53-induced phosphatase 1 that targets the Ser536 p65 subunit, which acts as a negative regulator of NF-κB signaling^46^.

IL-10 can be produced by macrophages as result of multiple signaling pathways and transcription factors after TLR activation^47^,^48^. Here we have demonstrated that PAF-induced potentiation of IL-10 production triggered by LPS is dependent on proteasome processing and NF-κB p105/50, but independent of CREB, STAT-3, and PPAR-γ. It has been demonstrated that NF-κB p50 homodimers are transcriptionally active and promote the production of IL-10^26^,^49^. The product of the *Nfκb1* gene, p105, originates NF-κB subunit p50 after proteasome processing, and functions as an IκB-like molecule by sequestering p50 in the cytoplasm^50^. After proteasome processing, p105 generates p50^48^. Additionally, macrophages from p105 KO mice fail to produce IL-10 when stimulated with LPS^51^,^52^. Hence, our results showing that PAF enhances LPS-induced p105 phosphorylation suggest, along with the fact that inhibition of p105/p50 prevented PAF-induced enhanced IL-10 production, that p105 has an essential role in mediating PAF-induced enhancement of IL-10.

It has been shown that the IKK complex activates the p65 subunit and regulates p105 phosphorylation^53^. More specifically, IKKα and IKKβ directly phosphorylate p105 on serine 927, which resides in a conserved motif homologous to the IKK target sequence in IκBα^54^,^55^, suggesting that PAF-mediated p105 activation could be dependent on the IKK complex. However, this needs to be further investigated.

Together, our data show that PAF interferes with selected pathways induced by TLRs in macrophages, driving them to a regulatory phenotype via IL10. PAF affects NF-κB activation by inhibiting p65 transcriptional activity and leading to a decrease in pro-inflammatory mediator formation and enhancement of IL-10 production in a manner dependent on the action of the NF-κB precursor p105. The regulation of TLR-mediated responses by PAF may provide potential new ways to manipulate the innate immune response.

## Additional Information

**How to cite this article**: Ishizuka, E. K. *et al*. PAFR activation of NF-κB p65 or p105 precursor dictates pro- and anti-inflammatory responses during TLR activation in murine macrophages. *Sci. Rep.* **6**, 32092; doi: 10.1038/srep32092 (2016).

## Supplementary Material

Supplementary Information
