# Supplementary material for: PAFR activation of NF-κB p65 or p105 precursor dictates pro- and anti-inflammatory responses during TLR activation in murine macrophages
Source: Sci Rep. 2016 Aug 24;6:32092. doi: 10.1038/srep32092 (PMC4995467; doi:10.1038/srep32092)

PAFR activation of NF-κB p65 or p105 precursor dictates pro- and anti-inflammatory responses during TLR activation in murine macrophages

Edson K. Ishizuka[1], Luciano Ribeiro Filgueiras[1], Francisco J. Rios[2], Carlos H. Serezani[3], Sonia Jancar[1*]

1 Department of Immunology, Institute of Biomedical Sciences, University of São Paulo, São Paulo, Brazil,

2 Institute of Cardiovascular and Medical Sciences, BHF Glasgow Cardiovascular Research Centre, University of Glasgow, Glasgow, United Kingdom,

3 Department of Microbiology and Immunology, Indiana University School of Medicine, Indianapolis, Indiana, United States of America (46202)

Supplemental Information

Figure 2A immunoblot

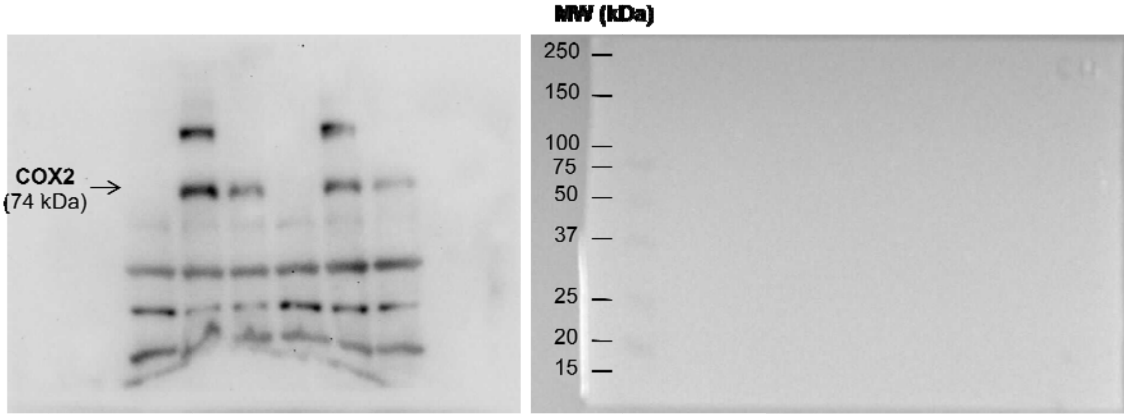

Figure 4A immunoblot

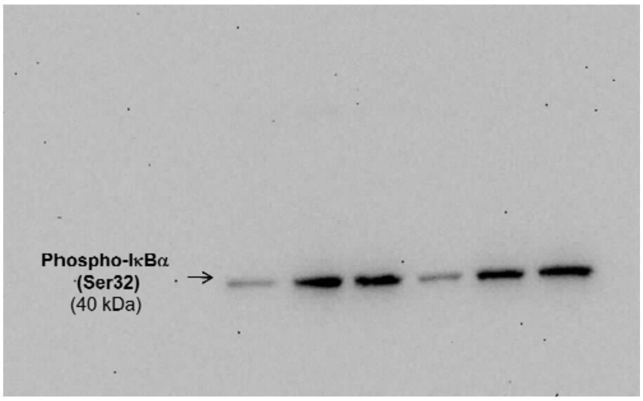

Figure 4B immunoblot

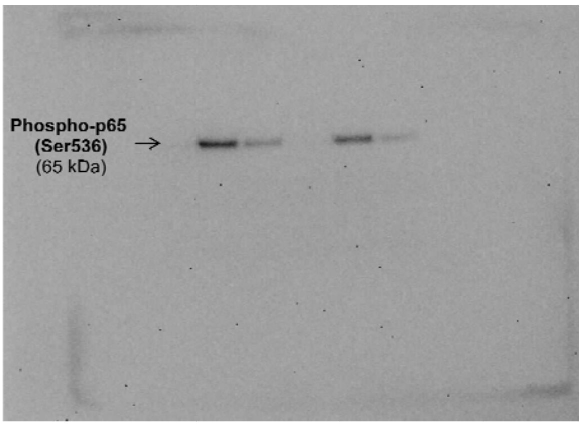

Figure 6B immunoblot

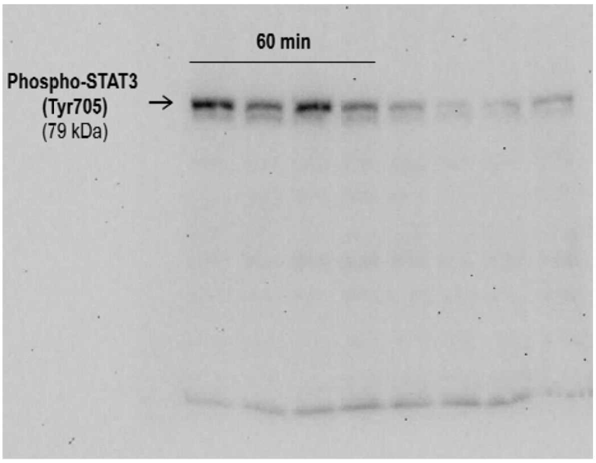

Figure 8B immunoblot

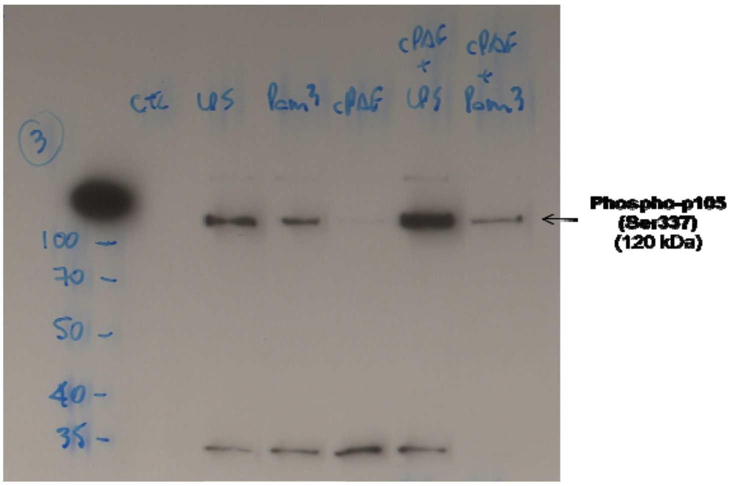

Supplement: Supplementary Information [file srep32092-s1.pdf]
